# Evaluation of the Acetone and Aqueous Extracts of Mature Stem Bark of *Sclerocarya birrea* for Antioxidant and Antimicrobial Properties

**DOI:** 10.1155/2012/834156

**Published:** 2012-05-17

**Authors:** Nicoline F. Tanih, Roland N. Ndip

**Affiliations:** ^1^Microbial Pathogenicity and Molecular Epidemiology Research Group, Department of Biochemistry and Microbiology, Faculty of Science and Agriculture, University of Fort Hare, Private Bag X1314, Alice 5700, South Africa; ^2^Department of Microbiology and Parasitology, Faculty of Science, University of Buea, Box 63, Buea, Cameroon

## Abstract

We assayed the antimicrobial activity of acetone and aqueous extracts of the stem bark of *Sclerocarya birrea* on some selected bacteria and fungi species including; *Streptococcus pyogenes, Plesiomonas shigelloides*, *Aeromonas hydrophila, Salmonella typhimurium, Cryptococcus neoformans, Candida glabrata, Trichosporon mucoides, *and *Candida krusei* using both agar well diffusion and minimum inhibitory concentration (MIC) assays. Based on the levels of activity, the acetone extract was examined for total polyphenolic content, radical scavenging and antioxidant activities. Total phenols of the extract were determined spectrophotometrically. The antioxidant activity was determined by the DPPH, ABTS and reducing power. All the bacteria and fungi species were susceptible to the plant extracts. The acetone extract was the most active for the bacterial species with MIC (0.156–0.625 mg/mL) while the aqueous extract was the most active for the fungi species with MIC (0.3125–1.25 mg/mL). The polyphenolic compounds were found as 27.2 mg/g tannic acid equivalent, 25.2 mg/g quercetin equivalent, 9.1 mg/g quercetin equivalent for phenols, flavonoid and flavonols respectively. The acetone extract exhibited a remarkable ability to scavenge radicals, strong reducing ability and a potential source of natural antioxidants. Both the acetone and aqueous extracts of *S. birrea* may provide a target for drug discovery.

## 1. Introduction

Awareness has increased recently on the possibility of health-related risk associated with oxidative stress [1, 2]. Oxidative stress initiated by highly reactive free radicals and oxygen species such as hydrogen peroxides, superoxide, lipid peroxyl, hydroxyl, nitric oxide, and peroxynitrite is present in biological systems from a wide variety of sources [3, 4]. The free radicals may oxidize nucleic acids, proteins, lipids, or DNA and cause degenerative diseases such as cancer, chronic inflammation, diabetes mellitus, atherosclerosis, myocardial infarction, arthritis, anemia, asthma, and neurodegenerative diseases [2, 4]. Inflammation, free radical damage, and oxidative stress are often the byproduct of normal cellular processes and are implicated in almost all debilitating degenerative conditions. Naturally, the human system has check-in mechanisms to deal with oxidative damage and free radical formation [2, 5]. These protective mechanisms maybe disrupted as a result of various pathological processes and thereby cause damage to the cells. Antioxidants have been reported to have significantly remedied this destructive effect [1]. 

Synthetic antioxidants used as food additives have been reported to be toxic both to humans and animals [5]. Presently, one of the most common used synthetic antioxidant is butylated hydroxytoluene (BHT) [2, 3]. This toxicity as well as general consumer rejection has led to decreasing use of synthetic antioxidants and geometric growth in research into naturally occurring products, particularly from medicinal plants, in search of alternative potent antioxidants. Much work has been done to find safe and potent natural antioxidants from various plant sources [4, 5].

Consequently, natural products such as plants and plant products have been the alternative target of natural antioxidants for food and pharmaceutical products lately based on their folkloric use in medicine since time immemorial. The presence of polyphenolic compounds such as flavonoids, phenols, flavonols, and proanthocyanidins in plants is associated with this antioxidant potential [4]. Synthetic antioxidants are generally compounds with phenolic structures of various degrees of alkyl substitution, whereas antioxidant bioactivity of medicinal plants is partly attributed to phenolic compounds [6]. Natural antioxidants that occur in medicinal plants act as freeradical scavengers and chain breakers, prooxidant metal ion complexes, and quenchers of singlet oxygen formation [7]. Antioxidant supplementation has been observed to be the most effective method to reduce oxidative stress. They retard the formation of toxic oxidation products, maintain nutritional quality, and increase shelf life [8].

*Sclerocarya birrea* is a medium-size-to-large deciduous tree with an erect trunk belonging to the family Anacardiaceae [9]. It is widely used for the treatment of several morbidities including proctitis, dysentery, and diarrhoea in South Africa and Africa at large [9–11]. Bark decoctions are used by the Xhosa and Zulu people as enemas for diarrhoea and the Vhavenda people for treating fevers, stomach ailments, and ulcers [9, 11]. Limited information exists on antioxidant activity of this plant. Mariod et al. [4] examined the antioxidant properties of methanolic extracts of different plant parts including the stem bark of *S. birrea* but no findings are recorded for the antioxidant activity of the acetone extract of the mature stem bark of *S. birrea *considering that successful isolation of biocompounds from plant material is largely dependent on the type of solvent used in the extraction procedure as well as the plant part [10].

 Microbial resistance to prevailing drugs remains an ever-growing challenge [12]. Bacterial species such as* Aeromonas hydrophila *(*A. hydrophila*)*, Salmonella typhimurium *(*S. typhimurium*), and* Plesiomonas shigelloides* (*P. shigelloides*) are Gram-negative organisms that cause gastrointestinal infections while *Streptococcus pyogenes (S. pyogenes*) is a Gram-positive organism that causes respiratory infections; they have all been reported to be resistant to a number of antibiotics [13, 14]. Furthermore, opportunistic fungi species like *Cryptococcus neoformans *(*C. neoformans*),* Candida glabrata *(*C. glabrata*),* Trichosporon mucoides *(*T. mucoides*)*, and Candida krusei *(*C. krusei*), which have been significantly associated with immune-compromised individuals have been noted to have an evolved resistance to drugs [10]. The biological activity of *S. birrea* has been described by several researchers but reports on the antimicrobial activity of the aqueous and acetone extracts of this plant on bacteria and fungi agents are limited to some species of microorganisms despite the universal usage of this plant in traditional medicine [9–11, 15]. This study was therefore aimed at examining the polyphenolic content and antioxidative potential of the acetone extract of mature stem bark of *S. birrea* as well as the bioactivity of the acetone and aqueous extracts on some bacteria and fungi species of medical importance as a guide for the valorisation and authentication of its use in complementary and alternative medicine.

## 2. Materials and Methods

### 2.1. Preparation of Plant Material

The stem bark of *S*. *birrea* was harvested from different trees at Nzhelele and transported to the University of Fort Hare. Identification of the plant was carried out by botanists at the School of Biological Sciences, University of Fort Hare, Alice with vouchers deposited at the school's herbarium (GEUFH01). The plant part was washed with tap water, chopped into small pieces, and dried at 40°C for one week in a hot air oven (Memment 854, Western Germany). The dried plant material was powdered using a blender (ATO MSE mix, England). Dried powdered plant material was further macerated in acetone and water, respectively, and crude extract of the plant was obtained as previously described [11].

### 2.2. Determination of Antimicrobial Activity of Mature Stem Bark of *S. birrea*

 The antimicrobial activity of the mature stem bark of *S. birrea* was evaluated against four reference strains of bacteria (*Streptococcus pyogenes* ATCC 49399, *Plesiomonas shigelloides* ATCC 51903, *Aeromonas hydrophila* ATCC 35654 and *Salmonella typhimurium *ATCC 13311), and fungi (*Cryptococcus neoformans *ATCC 66031, *Candida glabrata *ATCC 2001*, Trichosporon mucoides *ATCC 201382, and *Candida krusei *ATCC 14243), respectively. These organisms were selected based on their disease burden and increasing trend of antibiotic resistance in the developing world [13, 14, 16].

The agar well diffusion and broth microdilution methods were used to determine the antibacterial and antifungal activities of the acetone and aqueous extracts against the bacteria and opportunistic fungi [16]. Briefly, inocula of the bacteria and fungi species were prepared and adjusted to 0.5 McFarland turbidity standards. This was plated on Mueller Hinton (MH) agar for the bacterial species and potato dextrose agar for the fungi species. Inocula were spread uniformly on the plate and allowed to dry for 15 minutes. Wells (10 mm in diameter) were punched into the agar using sterile stainless steel borer and filled with 100 *μ*L of the extract at 100 mg/mL. Ciprofloxacin (0.05 *μ*g/mL) was used as positive control for the bacteria, while amphotericin B was used for the fungi species; 10% dimethyl sulfoxide (DMSO) was included in all experiments as negative controls. The plates were incubated at 37°C for 1 to 6 days depending on the organism (bacteria and fungi) after which diameters of zones of inhibition were measured in millimetres. The experiment was done in duplicates. Inhibitory activity of the plant was indicated by a clear zone of no microbial growth around each well.

### 2.3. Determination of Minimum Inhibitory Concentration

The minimum inhibitory concentration (MIC) assay was performed in 96 well plates [11]. Extract to be tested was prepared at a concentration of 10 mg/mL. Briefly, twofold serial dilution of the extract was carried out in the test wells in MH broth with concentration ranging from 0.156 to 10.00 mg/mL. Twenty microliter of an overnight broth culture of test organism was added to 180 *μ*L of extract-containing medium. Our controls were prepared with culture medium, bacterial suspension and broth only. Ciprofloxacin was used as positive control for the bacteria species while amphotericin B was used as positive control for the fungi species. ELISA plate reader (Model 680, Biorad, Tokyo, Japan) was used to measure the absorbance of the plates before and after one to six days of incubation at 37°C depending on the organism type. Absorbances were read at 620 nm and compared to check for microbial growth. The lowest concentration that inhibited the growth of the organism was considered as the MIC of the extract.

### 2.4. Polyphenolic Compounds

#### 2.4.1. Total Phenol

The phenolic content of the acetone extract was determined spectrophotometrically by the Folin Ciocalteu modified method [17]. Briefly, an aliquot of the extract (1 mL) was mixed with 5 mL of 10% Folin-Ciocalteu reagent and 4 mL of Na_2_CO_3_ (75% w/v). This mixture was vortexed for 15 s and incubated at 40°C for 30 min for colour development. The absorbance of the samples was measured at 765 nm (UV-VIS, Spectrophotometer Hewlett Packard, NJ, USA). The measurements were conducted in triplicate and the results reported as mean ± SD values. The result was expressed as mg/g tannic acid equivalent from the calibration curve.

#### 2.4.2. Total Flavonoids

Total flavonoid was estimated using the method of OrdoñEz [18]. This was based on the formation of a complex flavonoid-aluminium. A volume of 0.5 mL of 2% AlCl_3_ ethanol solution was added to 0.5 mL of extract solution. After one hour of incubation at room temperature, the absorbance was measured at 420 nm (UV-VIS Spectrophotometer Hewlett, Packard, NJ, USA). All determinations were done in triplicate, and values were calculated from calibration curve obtained from quercetin.

#### 2.4.3. Total Flavonols

Determination of total flavonol content was carried as previously described [19]. The reaction mixture consisted of 2.0 mL of the sample (acetone extract), 2.0 mL of AlCl_3_ prepared in ethanol, and 3.0 mL of sodium acetate (50 g/L) solution. The absorption at 440 nm was read after 2.5 h at 20°C. Total flavonoid content was calculated as quercetin (mg/g) equivalent from the calibration curve.

### 2.5. Determination of Reducing Power

The reducing power of the acetone extract was evaluated according to the method of Yen and Chen [20]. A volume of 1.0 mL of the extract prepared in distilled water and BHT and vitamin C (VIT C) (0–5.0 mg/mL) were mixed individually with the mixture containing 2.5 mL of 0.2 M phosphate buffer (pH 6.6) and 2.5 mL of potassium ferricyanide [K_3_Fe(CN)_6_] (1% w/v). The resulting mixture was incubated at 50°C for 20 min, followed by the addition of 2.5 mL of trichloroacetic acid (10% w/v), which was then centrifuged at 3000 rpm for 10 min. The upper layer of the solution (2.5 mL) was mixed with 2.5 mL of distilled water and 0.5 mL of ferrous chloride (0.1%, w/v). The absorbance was measured at 700 nm against a blank sample.

### 2.6. DPPH Radical Scavenging Activity

 The determination of scavenging activity of DPPH free radical in the acetone extract solution was executed using the method of Liyana-Pathiranan and Shahidi [21]. A solution of 0.135 mM DPPH in methanol was prepared, and 1.0 mL of this solution was mixed with 1.0 mL of extract prepared in methanol containing 0.025–0.5 mg of the plant extract and standard drugs separately (BHT and VITC). The reaction mixture was vortexed thoroughly and left in the dark at room temperature for 30 min. The absorbance of the mixture was measured spectrophotometrically at 517 nm as above. The ability of the plant extract to scavenge DPPH radical was calculated by the equation

(1) $$\begin{array}{cc} & \text{DPPH radical scavenging activity}\\ & \;=\left\{\left(\text{Abs control}-\text{Abs sample}\right)/\left(\text{Abs control}\right)\right\}\times 100, \end{array}$$

where Abs control is the absorbance of DPPH radical + methanol Abs sample is the absorbance of DPPH radical + sample extract or standard.

### 2.7. ABTS Radical Scavenging Activity

This was done using an antioxidant assay kit (Sigma, Germany, catalog no. CS0790). The principle of the antioxidant assay rests on the formation of a ferryl myoglobin radical from metmyoglobin and hydrogen peroxide, which oxidises the ABTS to produce a radical cation. The trolox working solution was prepared by mixing the trolox standard and 2.67 mL and 1x assay buffer. The reconstituted solution (1.5 mM) was used to prepare the trolox standard curve. Briefly, concentrations of trolox standard (0, 0.015, 0.045, 0.105, 0.21, and 0.42 nm) were prepared using assay buffer. In the wells of the trolox curve, 10 *μ*L of a trolox standard and 20 *μ*L of myoglobin working solution were mixed, while, in the wells of the test samples, 10 *μ*L of the test sample (acetone extract of *S. birrea*) and 20 *μ*L of myoglobin working solution were introduced and mixed. ABTS substrate working solution was added, the mixture incubated at room temperature, and the reaction abrogated using a stop solution. Absorbance was read at 405 nm.

### 2.8. Statistical Analysis

The experimental results were expressed as mean ± standard deviation (SD) of three replicates and were subjected to paired Student's *t*-test. Significant levels were tested at *P* < 0.05.

## 3. Results

### 3.1. Antimicrobial Activity of Crude Extracts

The acetone and aqueous crude extracts of *S. birrea* at the different concentrations (100 mg/mL and 50 mg/mL) demonstrated antimicrobial activity against all the microorganisms studied (Table 1). An inhibition zone diameter of ≥11 mm was chosen as a breakpoint for susceptibility [11]. *S. pyogenes *and *P. shigelloides* were the most susceptible organisms to all extracts while *Salmonella typhimurium* was the least susceptible with partial zones of inhibition. A zone diameter of inhibition of 27 ± 2.1 mm was recorded for *S. pyogenes* at 100 mg/mL. The acetone extract demonstrated good activity when compared to aqueous against all the bacterial species tested except for *Salmonella typhimurium* where the aqueous extract showed better activity at both concentrations (Table 1). Ciprofloxacin (0.025 mg/mL), the positive control, had a zone diameter of inhibition of 21–38 mm; the negative control (10% DMSO) showed no activity against our isolates. The zones of inhibition of the extracts and antibiotic were compared; no statistically significant difference was observed (*P* > 0.05).

For the fungi isolates, the aqueous extract demonstrated a better activity when compared to the acetone extract with the most susceptible organism being *C. neoformans* followed by *T. mucoides* with zone diameter of 25 ± 0.7 mm and 23 ± 2.9 mm, respectively, at 100 mg/mL while the least activity was reported for *C. krusei*. However, the acetone extract showed a better activity against *C. krusei *when compared to the aqueous extract at both concentrations.

### 3.2. MIC Determination

MIC of the extracts was determined against the organisms with ciprofloxacin as the positive control for the bacteria while amphotericin B was used for the fungi species. The acetone extract was the most active with regard to all the bacterial species tested presenting with overall smaller MIC values ranging from 0.156 to 0.625 mg/mL when compared to the aqueous extract, which had as MIC value 0.156 to 1.25 mg/mL. MIC value for the positive control, ciprofloxacin, ranged from 0.00396 to 0.156 mg/mL. Of the bacterial species tested, *Aeromonas hydrophila *was the most sensitive (Table 2). For the fungi species, MIC values ranged from 0.3125 to 1.25 mg/mL for the aqueous extract, which was less active when compared to the acetone extract (0.156 to 0.3125 mg/mL). The MIC of amphotericin B ranged from 0.025 to 0.625 mg/mL. *C. krusei *was the most sensitive of the fungi species tested (Table 2). However, there was no statistically significant difference between the MIC of the extracts and that of the control antibiotic (*P* > 0.05).

### 3.3. Polyphenolic Compounds

Since the acetone extract was more active than the aqueous extract, it was assayed for polyphenolic and antioxidant potential. The plant extract possessed high phenol contents (27.2 mg/g tannic acid equivalent) followed by flavonoid (25.2 mg/g quercetin equivalent) and flavonols (9.1 mg/g quercetin equivalent). Phenolic compounds, especially flavonoids and phenols, have been shown to possess significant antioxidant activity.

### 3.4. Antioxidant Activity

**Figure 1:**
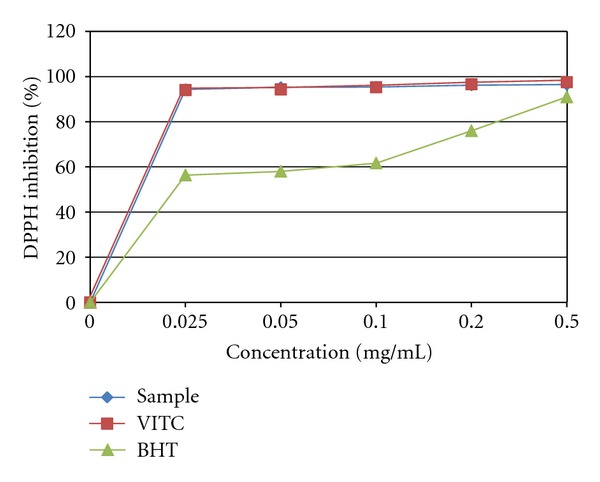
DPPH radical scavenging activity of the acetone bark extract of *S. birrea. *

The DPPH is a stable free radical that gives a strong absorption maximum at 517 nm emitting a purple colour. Absorbance decreases as a result of a colour change from purple to yellow when DPPH radical is reduced by hydrogen from a free radical scavenging antioxidant to form the reduced DPPH-H.  Figure 1 illustrates the DPPH radical scavenging activity of mature stem bark of *S. birrea *compared with VITC and BHT. The DPPH % inhibition increased as the plant extract concentration increased (0, 0.025, 0.05, 0.1, 0.2, and 0.5 mg/mL). As observed, the plant extract possessed tremendous DPPH radical scavenging activity. Interestingly, the activity of the plant was very similar to that of VITC but higher than that of BHT. 

**Figure 2:**
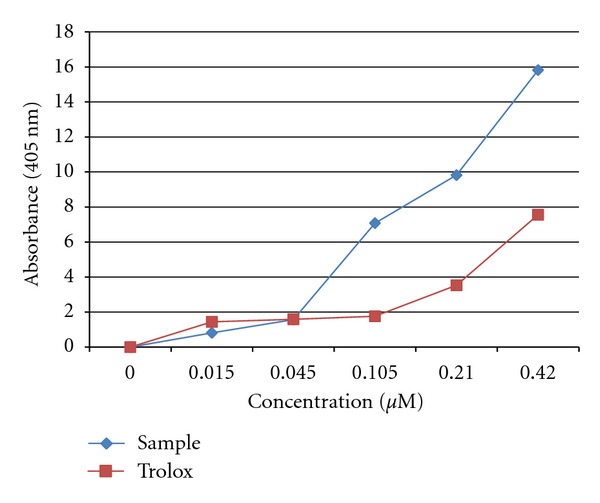
ABTS radical scavenging activity of the acetone bark extract of *S. birrea*.

In addition, antioxidant potency of the plant extract was further evaluated using ABTS (2, 2′azino-bis (3-ethyebenzthiazoline-6-sulfonic acid)).  Figure 2 shows the radical scavenging ability of the extract. As noted, an increase in the concentration of the extract (0.015, 0.045, 0.105 0.21, and 0.42 mg/mL) resulted in an increase of the absorbance. Our plant extract demonstrated a better antioxidant activity when compared to Trolox.

**Figure 3:**
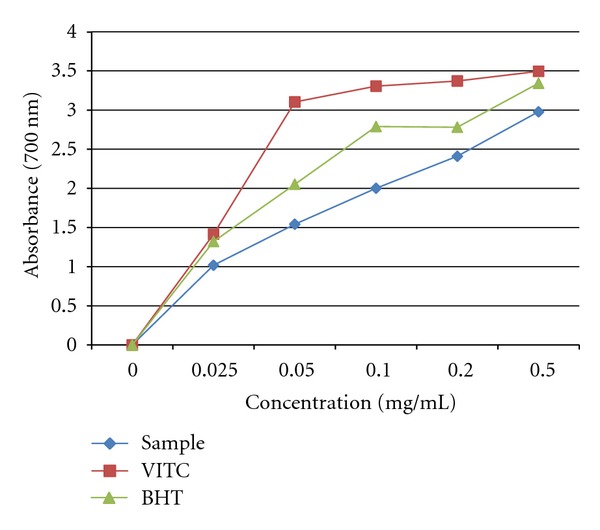
Total ferric reducing potential of acetone bark extract of *S. birrea*.

The plant extract was also evaluated for its ability to reduce iron (III) to iron (II) and compared with VITC and BHT standards (Figure 3). The reducing value of the extract was significantly lower than that of BHT and vitamin C. Generally, as the concentration (plant extract, VITC, and BHT) increased from 0 to 0.5 mg/mL, the absorbance also increased but at no point was the absorbance of plant extract higher when compared to the reference drugs.

## 4. Discussion

Phytochemical compounds in plants are known to be biologically active aiding as antioxidants and antimicrobials [9]. The problem of drug resistance seems an overwhelming challenge. There has been an increasing trend towards using medicinal plants to treat various diseases, especially in developing countries [22]. *S. birrea* has long been used in sub-Saharan Africa as a medicinal remedy for numerous ailments [9, 11] and it is believed to possess several therapeutic properties. The effect of acetone and aqueous extracts of mature stem bark was tested on some microbes and the largest inhibitory zone diameter was recorded for the acetone extract against all the bacterial strains compared with the aqueous extract implying that acetone could be a better solvent for extraction of phytochemicals (Table 1). The efficiency of acetone in the extraction of phytochemicals has been reported by some authors [9, 11]. The concentration 100 mg/mL demonstrated a better activity against the bacterial strains, though higher than 50 mg/mL which would be more beneficial as a prospective antimicrobial. Though *S. pyogenes *and *P. shigelloides *were the most susceptible of the bacteria evaluated, other bacterial species were susceptible to both the acetone and aqueous extracts. Our results are consistent with the findings of Eloff [9] who reported antimicrobial activity of both the bark and leaf extracts of *S. birrea* though not against the microorganisms we studied. Our results also corroborate the findings of Braca et al. [23] who documented plant parts of *S. birrea,* namely, the leaves, roots, and bark used to treat several conditions including diarrhoea, hypertension, diabetes, dysentery, and inflammations to have significant antimicrobial effect. For the fungi species, the aqueous extract demonstrated a better antimicrobial activity compared to the acetone extract with *C. neoformans* followed by *T. mucoides *being the most susceptible species. All fungi species were susceptible to both acetone and aqueous extracts again at a concentration of 100 mg/mL demonstrating better activity. This is in line with the finding of Masoko et al. [10] who found* S. birrea *to posses antifungal activity.

Interesting MIC values were observed for the different solvent extracts of *S. birrea *against the various bacterial species (Table 2). Ciprofloxacin used as the positive control inhibited growth of all the bacterial species (MIC 0.00396–0.156 mg/mL). There was no statistically significant difference between the MIC of extracts against bacterial species and that of ciprofloxacin (*P* > 0.05). The acetone extract was very active against all the tested pathogens except for *Salmonella typhimurium* where the aqueous extract showed a better activity. This is in line with the study of Eloff [9] who reported antibacterial activity of *S. birrea* leaves and bark extract against some Gram-positive and Gram-negative bacteria.

 Also, there was good activity of the different solvent extracts of *S. birrea* against the fungi species studied with no significant difference observed (*P* > 0.05). Amphotericin B used as positive control had an MIC of 0.025–0.0625 mg/mL. The aqueous extract of *S. birrea *was very active against all the tested pathogens except for *C. krusei* where the acetone extract showed a better activity. Our results are consistent with the findings of Hamza et al. [24] who reported inhibition of *C. glabrata*, *C. krusei,* and *Cryptococcus neoformans* by methanolic (an organic solvent) extracts of *S. birrea* roots. Based on MIC values, the acetone extract of mature stem bark of *S. birrea* would be a useful source for treating ailments caused by bacteria and fungi.

Polyphenols are the major plant compounds with high-level of antioxidant activity. Their ability to act as antioxidant rest on the fact that they are able to absorb, neutralize, and quench free radicals [7]. We evaluated the acetone extract of mature stem bark of *S. birrea *for its polyphenolic (flavonoids, flavonols, and phenols) contents and antioxidant and antimicrobial activities of both acetone and aqueous extracts on some selected microorganisms.

The plant extract possessed high polyphenolic content with phenols having the highest content followed by flavonoid and flavonols. Our results are in line with the report of Mariod et al. [4] who reported high total phenolic compounds in *S. birrea* bark although these authors did not identify if they used mature or young stem bark of *S. birrea *and the specific solvent used for extraction given that different solvents as well as plant parts may impact on the biological activity differently [10].

 The acetone extract demonstrated strong DPPH radical scavenging activity and ABTS antioxidant activity (Figures 1 and 2). Our plant demonstrated enormous activity, which was comparable to that of VITC a well-known standard antioxidant. These results are in line with the finding of Pretorius et al. [25] who reported that fruits harvested from *S. birrea* are rich in vitamin C, hence it is not surprising that the stem bark is rich in vitamin C. Interestingly, the antioxidant activity of our extract was better than that of BHT, a commercial antioxidant. However, Mariod et al. [4] reported antioxidant activity in the extract of *S. birrea* to be relative to that of BHA (butylated hydroxyanisole), a commercial antioxidant in foods. The scavenging activity of our extract was better than the Trolox standard in the ABTS assay. The high polyphenolic (flavonoids, flavones and phenols) content in the extract might be associated to the high-level antioxidant found with the DPPH and ABTS assays. The reducing ability of the acetone extract of *S. birrea* was determined by measuring the conversion of Fe^+3^ to Fe^+2^. The observed result showed that the extract possessed antioxidant activity in a concentration-dependent manner. This effect may suggest the ability of *S. birrea* to minimize oxidative damage to some vital tissues in the body [26].

DPPH radical is a model of a stable lipophilic radical. A chain reaction in lipophilic radical is known to be initiated by lipid autoxidation [27]. The antioxidant reacts with DPPH radical, reducing the number of DPPH radical molecules equal to the number of their available hydroxyl groups. Plant phenolics constitute one of the major groups of compounds acting as primary antioxidant or free radical terminators and initiating oxidation processes [4, 28]. Synergism of the polyphenolic compounds with each other in the plant extract may contribute to its antioxidant activity [29]. The differences in scavenging activity of the testing systems could be due to the different mechanisms involved in the radical-antioxidant reaction. For example, the solubility of the extracts in different testing systems, substrate used, and quantization method may influence the ability of herbs to quench different radicals [30]. As a result, it may be difficult to compare antioxidant activity based on antioxidant assay because of the different test system and the substrate to be protected [31].

In conclusion, polyphenolic compounds are present in the acetone extract of *S. birrea,* which could serve as a potential natural antioxidant. In addition, the extracts of this plant possess significant antibacterial and antifungal potential, which could provide an affordable and effective platform for newer drugs.

## Figures and Tables

**Table 1:** Zone of inhibition ± SD (mm) of the solvent extracts (mg/mL) of the stem bark of *S. birrea *against organisms.

Organism	Acetone		Aqueous	
	100	50	100	50
*S. pyogenes*	27 ± 2.1	25 ± 0.7	22 ± 0.9	20 ± 0.8
*A. hydrophila*	20 ± 0.0	17 ± 1.2	20 ± 0.0	17 ± 1.6
*P. shigelloides*	26 ± 4.9	23 ± 2.1	26 ± 2.8	22 ± 0.7
*S. typhimurium*	16 ± 1.3	14 ± 2.3	18 ± 2.9	16 ± 0.7
*C. neoformans*	15 ± 2.1	14 ± 1.6	25 ± 0.7	19 ± 0.8
*C. glabrata*	17 ± 1.2	15 ± 2.3	20 ± 0.0	18 ± 1.6
*T. mucoides*	20 ± 4.1	18 ± 2.2	23 ± 2.9	21 ± 0.8
*C. krusei*	16 ± 1.5	14 ± 2.1	14 ± 2.8	12 ± 0.7

**Table 2:** MIC values in mg/mL of acetone and aqueous extracts of mature stem bark of *S. birrea* including the positive controls (ciprofloxacin and amphotericin B) against the selected microorganisms.

Microorganism	Minimum inhibitory concentration (mg/mL)		Ciprofloxacin
	Acetone	Aqueous	
*S. pyogenes*	0.31250	0.62500	0.00396
*A. hydrophila*	0.15600	0.15600	0.01510
*P. shigelloides*	0.31250	1.25000	0.03120
*S. typhimurium*	0.62500	0.62500	0.15600
			Amphotericin B
*C. neoformans*	0.31250	1.25000	0.06250
*C. glabrata*	0.31250	0.62500	0.02500
*T. mucoides*	0.31250	0.62500	0.02500
*C. krusei*	0.15600	0.31250	0.03125
